# Low Intestinal *IL22* Associates With Increased Transplant-Related Mortality After Allogeneic Stem Cell Transplantation

**DOI:** 10.3389/fimmu.2022.857400

**Published:** 2022-04-29

**Authors:** Sakhila Ghimire, Katharina U. Ederer, Elisabeth Meedt, Daniela Weber, Carina Matos, Andreas Hiergeist, Florian Zeman, Daniel Wolff, Matthias Edinger, Hendrik Poeck, Wolfgang Herr, André Gessner, Ernst Holler, Sigrid Bülow

**Affiliations:** ^1^Clinic and Polyclinic for Internal Medicine III, University Hospital Regensburg, Regensburg, Germany; ^2^Institute of Clinical Microbiology and Hygiene, University Hospital Regensburg, Regensburg, Germany; ^3^Centre for Clinical Studies, University Hospital Regensburg, Regensburg, Germany; ^4^Leibniz Institute for Immunotherapy (LIT), Regensburg, Germany

**Keywords:** *IL22*, allogeneic SCT, GvHD, TRM, antibiotics, GPR41, GPR43

## Abstract

The role of IL-22 in adult patients undergoing allogeneic stem cell transplantation (SCT) is of major interest since animal studies showed a protective and regenerative effect of IL-22 in graft versus host disease (GvHD). However, no clinical data exist on the tissue expression. Here we demonstrate that patients not suffering from transplant-related mortality (TRM) show significantly upregulated *IL22* expression during histological and clinical GI-GvHD (p = 0.048 and p = 0.022, respectively). In contrast, in GvHD patients suffering from TRM, *IL22* was significantly lower (p = 0.007). Accordingly, lower *IL22* was associated with a higher probability of TRM in survival analysis (p = 0.005). In a multivariable competing risk Cox regression analysis, low *IL22* was identified as an independent risk factor for TRM (p = 0.007, hazard ratio 2.72, 95% CI 1.32 to 5.61). The expression of *IL22* seemed to be microbiota dependent as broad-spectrum antibiotics significantly diminished *IL22* expression (p = 0.019). Furthermore, *IL22* expression significantly correlated with G-protein coupled receptor (GPR)43 (r = 0.263, p = 0.015) and GPR41 expression (r = 0.284, p = 0.009). In conclusion, our findings reveal an essential role of *IL22* for the prognosis of patients undergoing allogeneic SCT.

**Graphical Abstract:**
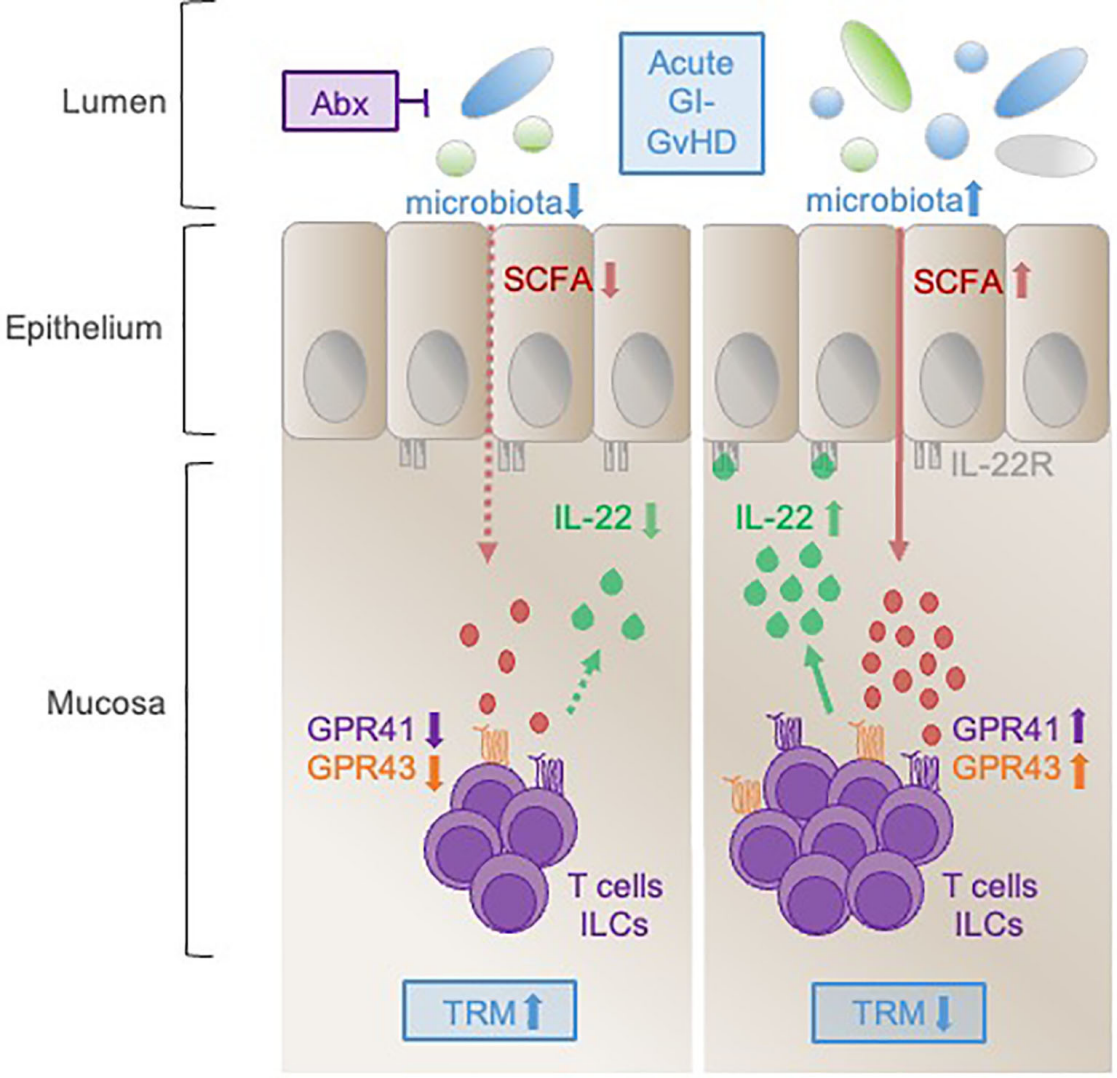
After allogeneic SCT, IL-22 producing T cells and ILCs infiltrate into the mucosa of the adult GI-tract. Optimal IL-22 induction is influenced by intact microbiota *via* short chain fatty acids (SCFAs) stimulating the G-protein coupled receptors GPR41 and GPR43. In contrast, the use of broad-spectrum antibiotics abrogates IL-22 expression by suppression of commensal bacteria and impairment of the consecutive SCFA-GPR axis. During GvHD, upregulation of IL-22 favors survival whereas IL-22 deficiency associates with increased TRM.

## Introduction

Allogeneic hematopoietic stem cell transplantation (SCT) is a potentially curative therapy for patients with hematological disorders. Nevertheless, SCT is still associated with substantial mortality and morbidity with up to 50% of patients at the risk of developing acute graft versus host disease (GvHD) (1–3). Acute GvHD affects the skin, liver, and the gastro-intestinal (GI) tract (2). Severe GI-GvHD is associated with increased transplant-related mortality (TRM), reduced survival, and impaired quality of life (4). Within the GI-tract, epithelial cells are a sensitive target of GvHD. The gut epithelium is composed of enterocytes, Paneth cells, goblet cells, enteroendocrine cells, and tuft cells which are crucial players in tissue homeostasis (5). Thereby, the epithelial cell layer segregates gut microbiota from host immune cells and mediates signals between gut microbes and host immune cells (5). Protection and maintenance of gut epithelium remain crucial in the prevention and treatment of GI-GvHD.

IL-22 was first discovered in early 2000 and was described as IL-10 related T cell-derived inducible factor (6, 7). The human *IL22* gene is located on chromosome 12q15, close to the *IL26* and *INFγ* genes, and shares 80.8% sequence homology with murine IL-22 (8). In lymphoid tissue, αβ T cells, γδ T cells, innate lymphoid cells (ILCs), and natural killer T cells have been identified as cellular sources of IL-22 (9). In the context of GvHD, especially intestinal IL-22 produced by group 3 ILCs (ILC3s) has been shown to support intestinal epithelial regeneration and barrier function (10, 11). In murine models of acute GvHD, treatment with recombinant IL-22 increased the number of stem cells, improved epithelial integrity, enhanced the expression of antimicrobial peptides such as Reg3β and Reg3γ; and finally resulted in reduced mortality (11, 12). Furthermore, IL-22 has been shown to stimulate mucus production in a STAT3 dependent manner and goblet cell reconstitution within the mucosal epithelium leading to rapid attenuation of inflammation in a model of ulcerative colitis (13).

Concerning microbiota-induced IL-22 expression in murine models, Yang and colleagues showed that microbiota-derived short-chain fatty acids (SCFA) promoted IL-22 production by ILCs and T cells through G-protein coupled receptor 41 (GPR41) by inhibiting histone deacetylase (14). In addition, Chun et al. demonstrated that GPR43 regulates ILC3 expansion and IL-22 expression *via* AKT and STAT3 axis, thus supporting the role of microbiota in IL-22 expression (15).

Given the beneficial role of IL-22 in the context of inflammation, we sought to analyze the expression of *IL22* in the gastrointestinal tract of SCT patients in the course of GvHD development. We found an association of high intestinal *IL22* levels with low TRM after allogeneic SCT emphasizing a protective role of IL-22. Of importance, the use of broad-spectrum antibiotics strongly suppressed *IL22* expression, and *IL22* correlated with *GPR41* and *GPR43* expression. Our findings imply that enhancing expression or treatment with IL-22 has great potential to attenuate TRM after allogeneic SCT.

## Materials and Methods

### Patient Characteristics

Biopsies of 118 patients receiving an allogeneic SCT at the University Hospital Regensburg, Germany, from 2008 to 2016 were included in the study. All patients gave informed consent for the GI-tract biopsies. Patient characteristics are given in **Table 1**. The study was approved by the local ethical review board of the University of Regensburg (approval numbers: 02/220, 09/059, and 17-619-101).

**Table 1:** Summary of patient characteristics.

Characteristics		mean	(range)
**Age in years**			
	Patients (N = 118)	53	(20-69)
**Sex**		**n**	**(%)**
	male	66	55.9
	female	52	44.1
**Diagnosis**			
	Acute leukemia	67	56.8
	Myelodysplastic syndrome	14	11.9
	Myeloproliferative syndrome	7	5.9
	Lymphoma	30	25.4
**Stage of underlying disease**			
	Early	34	28.8
	Intermediate	44	37.3
	Advanced	40	33.9
**Donor type**			
	Unrelated donor	74	62.7
	Sibling	38	32.2
	Haploidentical donor	6	5.1
**Stem cell source**			
	PBSC	110	93.2
	BM	8	6.8
**Conditioning regimen**			
	Reduced intensity	104	88.1
	Standard	14	11.9
**Immunosuppression**			
	CyA/MTX	96	81.4
	CyA/MMF	13	11.0
	Tacro/MMF	3	2.5
	PostTxCy/Tacro/MMF	6	5.1
**Clinical GI-GvHD stage at time of biopsy**			
	No GvHD	60	50.8
	Stage 1	32	27.1
	Stage 2	11	9.3
	Stage 3	8	6.8
	Stage 4	7	5.9
**Lerner GI-GvHD stage at time of biopsy**			
	No GvHD	56	47.4
	Grade 1	36	30.5
	Grade 2	11	9.3
	Grade 3	9	7.6
	Grade 4	6	5.1

PBSC, peripheral blood stem cells; BM, bone marrow; CyA, cyclosporin; MTX, methotrexate; MMF, mycophenolate mofetil; Tacro, tacrolimus; PostTxCy, post-transplant cyclophosphamide.

### Biopsy Characteristics

Gastro-intestinal biopsies were obtained either in the course of screening study in asymptomatic patients (median 30 days, range 13 - 2,345 days), because of clinical symptoms indicative of *de novo* onset (median 51 days, range 14 - 479 days), or during persistence or recurrence of acute GI-GvHD (median 130 days, range 37 - 538 days). Biopsies were histologically graded for the acute GI-GvHD according to the Lerner’s grading system (16). There were 56 patients who had no histological signs of acute GI-GvHD, 36 patients had GvHD grade 1, 11 patients had GvHD grade 2, 9 and 6 patients had GvHD grade 3 and 4, respectively.

### RNA Extraction, cDNA Synthesis and qPCR

Following retrieval, biopsies were immediately transferred to 500 µl RNA protect (Qiagen) and were stored at - 80°C until RNA extraction. RNA extraction, including DNA digestion, was performed with RNeasy Mini Kit (Qiagen) as per the manufacturer’s instructions. RNA concentration and purity were monitored with NanoDrop and Bioanalyser, respectively. One microgram of RNA was transcribed to cDNA using moloney murine leukemia virus (MMLV) reverse transcriptase (Promega) according to the manufacturer’s instructions. qPCR was performed on a Mastercycler Ep Realplex (Eppendorf) for *GPR43* and *GPR41* and on HT 7900 Real-Time PCR system (Applied Biosystems) for *IL22* using QuantiFast SYBR Green PCR Kit (Qiagen). Genes of interest were normalized to the *18S rRNA* reference gene.

Following gene-specific primer pairs were used:

*IL22*, forward: 5’-AGC-CCT-ATA-TCA-CCA-ACC-GC-3’, reverse: 5’-TCT-CCC-CAA-TGA-GAC-GAA-CG-3’; *GPR43*, forward: 5’- GTA-GCT-AAC-ACA-AGT-CCA-GTC-CT -3’, reverse: 5-CTA-GGT-GTT-GCT-TTG-AAG-CTT-GT -3’; *GPR41*, forward: 5’- GCC-AAC-TGC-ACT-AGG-TCT-GGA-GAG-3’, reverse: 5’-CTT-GCC-CAC-GAA-GAC-CAC-CA-3’; *18S rRNA*, forward: 5’-ACC-GAT-TGG-ATG-GTT-TAG-TGA-G-3’, reverse: 5’-CCT-ACG-GAA-ACC-TTG-TTA-CGA-C-3’.

### Immunofluorescence

Formalin-fixed paraffin embedded (FFPE) biopsies were cut approximately 3 µm thick and were immersed in xylene and descending alcohol line. Antigen retrieval was performed with citrate buffer (pH 7.2) at 350 watts for 32 minutes in a microwave. Subsequently, the biopsy was blocked with 20% bovine serum albumin (BSA) for 20 minutes, rinsed three times with PBS followed by incubation with anti-IL-22 (mouse monoclonal, clone 2D5, Merck Millipore) and anti-CD3 (rabbit monoclonal, clone SP7, Thermo Scientific) primary antibodies at 1:50 dilution in 1% BSA for 1 hour. Primary antibodies were labeled with Alexa Flour (AF) 488 (for IL-22) and AF594 (for CD3) and secondary antibodies for another hour. Nuclei were counterstained with DAPI. Images were taken with a Zeiss epifluorescence microscope.

### Statistical Analyses

Data were analyzed in SPSS version 26 (IBM) and R version 4.1.2 (The R Foundation for Statistical Computing). Test of normality was performed using Shapiro-Wilk test. Normally distributed data were analyzed with t-test or one way ANOVA. Non-normally-distributed data were analyzed with Mann-Whitney *U* test or Kruskal-Wallis test. Correlations were assessed by using Pearson or Spearman-rho correlation coefficient for normal and non-normally distributed data, respectively. Kaplan-Meier method was used to calculate the probability of TRM. To analyze the impact of *IL22* on TRM, uni- and multivariable competing risk Cox regression models were used accounting for relapse related mortality as competing risk event. Time to event was defined from day of biopsy to day of death or to last day of patient being confirmed to be alive (censored cases). The multivariable model was adjusted for the additional covariates GI-GvHD, age, steroids, stage of disease, donor type, broad spectrum antibiotics and conditioning. Since biopsies were taken at different time points after SCT, we also added time from SCT to biopsy as potential confounder to the model. Hazard Ratios (HR) and corresponding 95%-confidence intervals (95%-CI) are reported as effect estimates. All competing Cox regression analyses were evaluated using the CumIncidence function (17) and the factor2ind function (18) of the cmprsk_2.2-11 package in R version 4.1.2. Multivariable competing risk regression analyses was done in 110 of 118 patients since data on antibiotics at the time of biopsy were missing on 8 patients. A p-value < 0.05 was considered statistically significant for all analyses. Data were depicted using Graph Pad Prism, version 7.00 (GraphPad Software).

## Results

### *IL22* Is Upregulated in Patients Surviving GvHD

**Figure 1:**
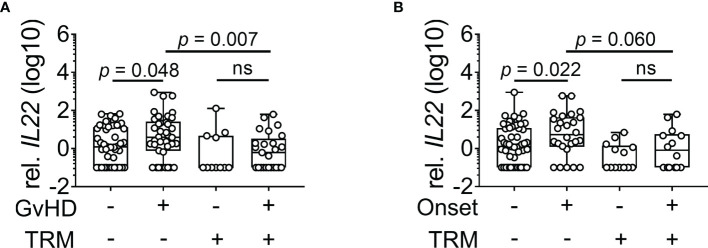
*IL22* expression in dependence of GvHD and TRM. Patients were grouped according to absence or presence of TRM. *IL22* gene expression normalized to *18s rRNA* was measured. **(A)** Expression of *IL22* in patients during histological GI-GvHD (no TRM: n = 82, TRM: n = 36). **(B)** Expression of *IL22* in patients without and during clinical onset of GvHD (no TRM: n = 78, TRM: n = 27). Box plot diagrams depict median, upper, and lower quartiles and whiskers indicate minimal and maximal values. Negative values were set to 0.1. Statistical testing was performed using Mann-Whitney *U* test. ns, not significant.

First, we were interested in whether GvHD patients show distinct *IL22* mRNA expression compared to patients who did not develop acute GI-GvHD. When we classified patients according to Lerner grades, *IL22* expression did not significantly differ between patients without GvHD or with mild to severe GvHD (**Supplementary Figure 1A**). When comparing screening biopsies of patients without clinical GI-GvHD to those derived at clinical onset, significant upregulation of *IL22* was found in the latter (*p* = 0.043, **Supplementary Figure 1B**). Since IL-22 administration has a positive impact on survival in murine GvHD (11, 12), we tested the hypothesis that *IL22* expression might have an impact on transplant-related mortality (TRM) and therefore separated the patients in regard to both, GvHD and TRM. In our cohort, patients experiencing TRM died mainly of infection, GvHD, or toxicity. The precise cause of death is listed in **Table 2**. Most importantly, within the patient group suffering from acute, histologically proven GI-GvHD, those with TRM showed significantly lower *IL22* mRNA expression compared to those without TRM (*p* = 0.007, **Figure 1A**). Similarly, clinically defined onset patients tended to show lower *IL22* expression in the TRM group as compared to the non-TRM group (p = 0.060, **Figure 1B**). Interestingly, in the patient group without TRM, histological GvHD was associated with a significantly higher *IL22* expression compared to patients without histological GvHD (*p* = 0.048, **Figure 1A**). From a clinical perspective, in the patient group without TRM, *IL22* expression was pronounced at the onset of GvHD compared to screening biopsies (*p* = 0.022, **Figure 1B**). In conclusion, *IL22* is significantly upregulated in GvHD patients not dying from TRM.

**Table 2:** Cause of death compounded as TRM.

Cause of Death (COD)	Frequency	%
Infection and GvHD	12	33.4
Infection	10	27.8
GvHD	7	19.4
Toxicity	4	11.1
Infection and graft failure	2	5.5
Secondary malignancy	1	2.8
Total	36	100

### *IL22* Expression Is Low in Patients With Subsequent TRM

**Figure 2:**
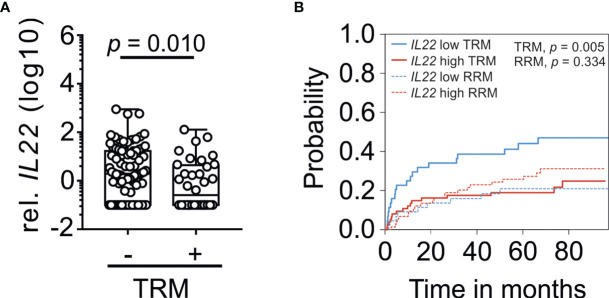
*IL22* expression with respect to TRM. **(A)** *IL22* gene expression in patients grouped according to absence or presence of TRM without further subgrouping (non-TRM: n = 82, TRM: n = 36). *IL22* gene expression was normalized to *18s rRNA*. Box plot diagrams depict median, upper, and lower quartiles and whiskers indicate minimal and maximal values. Negative values were set to 0.1. Statistical testing was performed using Mann-Whitney *U* test. **(B)** Association of *IL22* with cumulative incidence function estimates for competing risk data. Patients were dichotomized in accordance to high (red line) and low expression (blue line) of *IL22* based on Youden index. The cumulative risk of TRM and RRM in dependence of the months after biopsy is shown.

When we examined the impact of *IL22* on prognosis after allogeneic SCT, including patients with and without GvHD, we observed significantly lower *IL22* expression in the TRM group compared to the non-TRM group (*p* = 0.010, **Figure 2A**). To further address this association, we dichotomized patients into high and low expression of *IL22* based on ROC curve analysis and a Youden index of 0.6435. Kaplan-Meier analysis revealed a significantly higher probability of TRM in patients with low *IL22* expression (*p* = 0.004, **Supplementary Figure 2**). In addition to the 36 patients dying by TRM, 31 patients died of relapse (relapse related mortality, RRM) as a competing event. Moreover, observation times, as measured from biopsy retrieval until the occurrence of the event (such as TRM or relapse) or the last follow up, varied. Therefore, a time-dependent competing risk analysis was performed revealing a significant association of low *IL22* with the probability of TRM (*p* = 0.005, **Figure 2B**). In contrast, no significant association of *IL22* and RRM was observed (*p* = 0.334, **Figure 2B**). Time-dependent Cox regression analysis revealed low *IL22* (*p* = 0.007, hazard ratio 2.72, 95% CI 1.32 to 5.61) and acute GI-GvHD (*p* = 0.010, hazard ratio 3.12, 95% CI 1.32 to 7.39) as independent risk factors for TRM (**Table 3**). Established risk factors such as age, steroids, stage of disease, donor type, broad spectrum antibiotics or conditioning were not significantly associated. Furthermore, no influence of the time between the recent transplantation and biopsy retrieval was found. In summary, low expression of *IL22* in intestinal biopsies associates with an increase in transplant-related mortality after allogeneic stem cell transplantation.

**Table 3:** Multivariable Cox regression analysis for TRM.

Risk factors	P value	HR	95% CI for HR
Low *IL22* (n = 41)	0.007	2.72	1.32 – 5.61
Grade 2-4 GI-GvHD (n = 25)	0.010	3.12	1.32 – 7.39
Patient’s age (> 50 years, n = 75)	0.140	1.93	0.80 – 4.66
Steroid (n = 59)	0.170	1.81	0.77 – 4.26
Broad spectrum antibiotics (n = 48)	0.340	1.40	0.71 – 2.78
Conditioning (Standard, n = 13)	0.470	1.41	0.55 – 3.61
Donor (MUD, n = 68)	0.750	0.88	0.42 – 1.88
Stage of underlying disease (advanced, n = 37)	0.750	1.13	0.53 – 2.40
Time of recent SCT to biopsy	0.970	1.00	1.00 – 1.00

A total of 110 patients were analyzed. High-risk groups with respective numbers are indicated for categorical variables. HR, hazard ratio; CI, confidence interval; MUD, matched unrelated donor.

### Broad-Spectrum Antibiotics Suppresses *IL22* Expression

**Figure 3:**
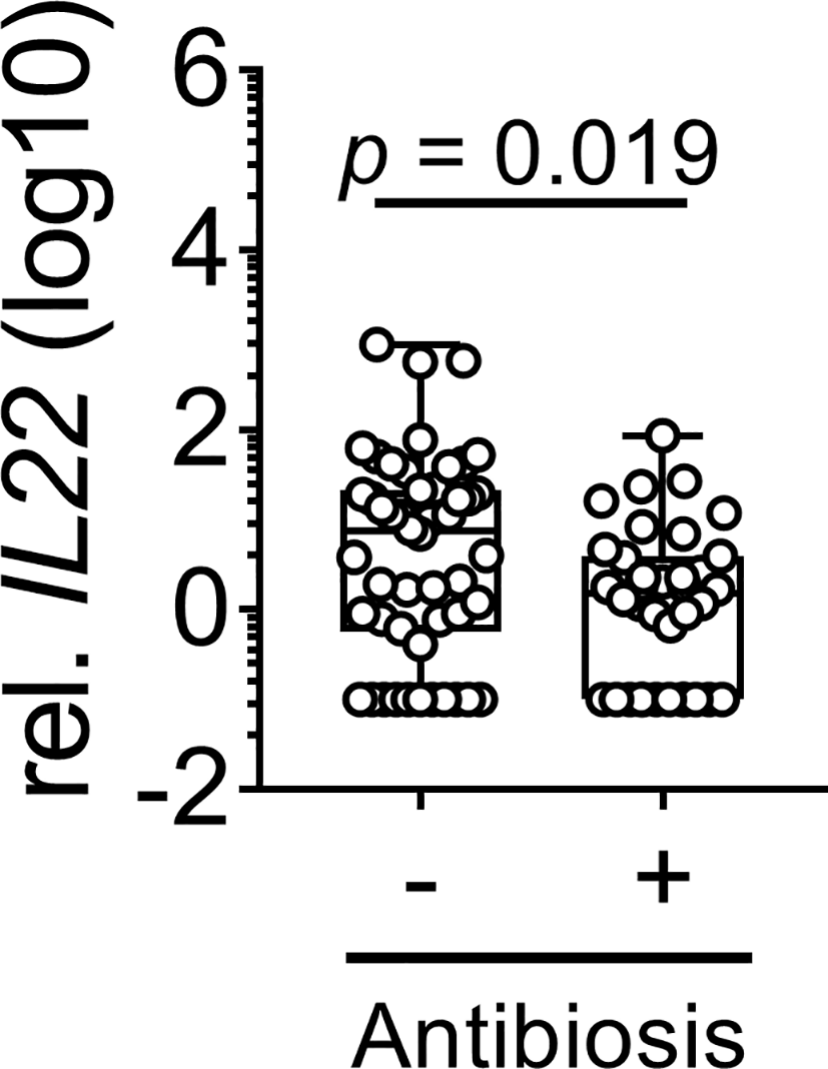
Impact of broad-spectrum antibiotics on *IL22* expression. *IL22* expression in biopsies derived from the lower GI tract of patients after allogeneic SCT is depicted (no Abx: n = 50, Abx: n = 36). Box plot diagram depicts median, upper, and lower quartiles and whiskers indicate minimal and maximal values. Negative values were set to 0.1. Statistical testing was performed using Mann-Whitney *U* test.

The application of broad-spectrum antibiotics (Abx) within 7 days before obtaining biopsies was used as an indirect indicator of microbiota damage (19) with 60% of patients receiving carbapenems (meropenem), 35% of patients receiving piperacillin/tazobactam and only 5% of patients receiving cephalosporins (ceftazidim). Since the lower GI-tract harbors the majority of microbiota (20), we analyzed *IL22* status in lower GI-tract (n = 86) with respect to use of Abx treatment and observed a significant lower *IL22* mRNA in patients receiving Abx compared to patients not treated with Abx (*p* = 0.019, **Figure 3**). When we included upper GI tract biopsies in our analyses, we still observed marked suppression of *IL22* in antibiotic group with a trend towards significance (no Abx: median = 1.88, range = 0 - 898; Abx: median = 1.21, range = 0 - 85.8, *p* = 0.085). In conclusion, reduction of commensals as implicated by the use of Abx negatively affected *IL22* expression in the lower GI in our cohort.

### *IL22* Correlates With GPR41 and GPR43 Expression

**Figure 4:**
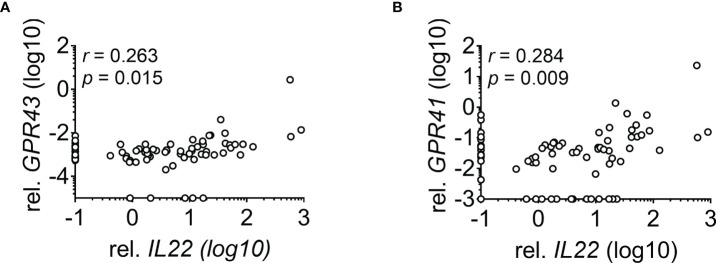
Correlation between *IL22* and *GPRs.* Correlation of *IL22* with **(A)** *GPR43* (n = 85) and **(B)** *GPR41* (n = 84) in biopsies derived from the lower GI tract is depicted. Negative values for *IL22* were set to 0.1, for *GPR43* to 0.00005 and for *GPR41* to 0.001. Statistical testing was performed using Spearman`s correlation.

To identify IL-22 cellular source in patient biopsies, we performed immunofluorescent staining using IL-22- and CD3-specific antibodies in sigmoid colon biopsies (**Supplementary Figure 3**). IL-22^+^ cells were detected both within CD3^+^ and CD3^-^ cells. Microbiota-derived SCFA were shown to increase IL-22 expression in CD3^-^ ILC3 *via* GPR43 and both CD3^-^ ILCs and CD3^+^ T cells *via* GPR41 in murine models (14, 15). To further explore the role of microbiota on *IL22* expression in allogeneic SCT patients, we examined the association of *IL22* with SCFA receptors GPR43 and GPR41 in the intestinal biopsies of the lower GI tract (**Figure 4**). Indeed, a significant positive correlation between *IL22* and *GPR*s was observed.

## Discussion

The central goal of this study was to investigate the role of *IL22* in the context of allogeneic SCT with focus on GI-GvHD. Here, we show for the first time a significant reduction of *IL22* in the intestinal biopsies of patients who experienced TRM emphasizing the importance of IL-22 in the context of stem cell transplantation. Especially increased expression of *IL22* in non-lethal compared to lethal GI-GvHD implicates IL-22 as a major survival factor in patients suffering from GvHD. In this context, the upregulation of *IL22* appears to compensate or counter-regulate ongoing inflammation as already seen for Foxp3 (21), IDO (22) and GPR (23) in gastric or colonic biopsies of GvHD patients. A previous study by Lounder and colleagues analyzed serum IL-22 in pediatric SCT patients and showed an association with higher serum IL-22 with subsequent GI-GvHD but not with the incidence of TRM (24). In contrast, we found a significant reduction of intestinal *IL22* in patients with subsequent TRM. Thereby, differences between a pediatric and adult cohort might exist. Moreover, intestinal *IL22* mRNA measured in our study might not inevitably reflect IL-22 protein and, additionally, IL-22 levels in the gut and in the serum do not necessarily have to correlate. In this context, data exist on Reg3α where high Reg3α levels in the serum paradoxically reflected the lack of Reg3α-positive Paneth cells in the gut mucosa (25, 26).

The observation that low intestinal *IL22* expression is associated with a higher rate of TRM implicates the potential role of IL-22-mediated epithelial regeneration that has previously been described in the murine model of GvHD (10). In line with these data, the multivariable Cox regression model revealed low *IL22* levels as a possible risk factor for TRM, which, interestingly, was independent of the presence of GvHD at the time of biopsy. The association of *IL22* with cumulative incidence of TRM in a competing risk analysis makes IL-22 an attractive tool for therapy in transplantation. In fact, the administration of recombinant IL-22 in a murine GvHD model strongly decreased gut GvHD pathology (27). In a recent phase II clinical trial, the use of IL-22 (in combination with standard immunosuppressants) for the treatment of acute GvHD showed a positive response rate of 100%, 75%, and 58% in a low-, intermediate-, and high-risk biomarker constellations, respectively (28), providing a proof-of-concept for the efficacy of IL-22 therapy. It may be hypothesized that IL-22 plays important role in the recovery from treatment-associated tissue and stem cell damage and, thus, increases tissue tolerance and enhances survival.

One remarkable finding of our work is the influence of Abx on *IL22* expression in intestinal biopsies. Owing to the detrimental effect of Abx on microbial diversity (19, 29, 30), butyrate-producing bacteria (31) and the receptors of microbial metabolite such as GPR109a and GPR43 (23), our data hints towards the necessity of balanced microbiota, metabolites, and receptors for optimal *IL22* production in GvHD patients. In analogy to data in mice (15), Abx treatment in patients with loss of commensals could lead to a reduction of protective metabolites followed by downregulation of SCFA receptors and, hence, reduced *IL22*. Besides T cells, ILCs are the main producer of IL-22 during inflammation (32). ILCs are known to express GPR43 to a higher extent when compared to macrophages, dendritic cells, or NK cells in the murine colon (15). In line with this observation, we found a significant positive correlation between *IL22* and *GPR43* in the intestinal biopsies of patients. Additionally, *IL22* also correlated significantly with *GPR41* which was shown to promote SCFA-mediated IL-22 production (14). Thus, our data suggest that the protective role of IL-22 is a possible explanation for the impact of microbiota on outcome after allogeneic SCT. Although the decrease of *IL22* in the course of Abx use and correlation of *IL22* with *GPR*s strengthen this hypothesis, a limitation of our study is the lack of data on the microbiome status at the time of biopsy retrieval. Therefore, the relation between *IL22* expression and microbiota status will be addressed in future studies.

In conclusion, our analysis of intestinal biopsies from allogenic SCT patients further highlights the outstanding importance of IL-22 on the clinical outcome after allogeneic SCT and strengthens the therapeutic potential of IL-22 for the amelioration of GvHD associated complications.

## Data Availability Statement

The raw data supporting the conclusions of this article will be made available by the authors, without undue reservation.

## Ethics Statement

The studies involving human participants were reviewed and approved by Aktive Ethikvoten der Ethik-Kommissionan der Universität Regensburg Email: ethikkomission@ur.de. The patients/participants provided their written informed consent to participate in this study (approval no: 02/220, 09/059, 17-619-101). The patients/participants provided their written informed consent to participate in this study.

## Author Contributions

SG performed experiments, collected and analyzed data, and wrote the manuscript. KE performed experiments, collected data, and revised the manuscript. EM and DWe collected clinical data and revised the manuscript. CM, AH, FZ, DWo, ME, HP, WH, AG, EH discussed and revised the manuscript. SB supervised the project, analyzed data, and wrote the manuscript. All authors contributed to the article and approved the submitted version.

## Funding

This work was funded by the Deutsche Forschungsgemeinschaft (DFG, German Research Foundation) -Projektnummer 324392634 - TRR 221”, Wilhelm Sander Foundation, Grant 2017.020.1 “Dysbiosis and intestinal immunoregulation in GvHD”, Marie Curie Initial Training Networks, Project Number 315963, the Else-Kroener-Fresenius-Stiftung, the Bavarian Ministry of Science and the Arts in the framework of the Bavarian Research Network ‘New Strategies Against Multi-Resistant Pathogens by Means of Digital Net-working – bayresq.net and CRC 1371, DFG “Microbiome signatures”.

## Conflict of Interest

The authors declare that the research was conducted in the absence of any commercial or financial relationships that could be construed as a potential conflict of interest.

## Publisher’s Note

All claims expressed in this article are solely those of the authors and do not necessarily represent those of their affiliated organizations, or those of the publisher, the editors and the reviewers. Any product that may be evaluated in this article, or claim that may be made by its manufacturer, is not guaranteed or endorsed by the publisher.

## Supplementary Material

Supplementary Figure 1 *IL22* expression in dependence of GvHD *IL22* gene expression normalized to *18s rRNA* was measured. **(A)** Patients were grouped according to histological GI-GvHD using Lerner`s grading system (no GvHD: n = 56, GvHD 1: n= 36, GvHD 2: n= 11, GvHD 3-4: n = 15), **(B)** Expression of *IL22* in patients during clinical GvHD (screen: n = 65, onset: n = 40, ongoing: n = 13). Box plot diagrams depict median, upper, and lower quartiles and whiskers indicate minimal and maximal values. Negative values were set to 0.1. Statistical testing was performed by Mann-Whitney *U* test. ns, not significant.

Supplementary Figure 2 Association of *IL22* with probability of TRM. Patients were dichotomized in accordance to high (red line) and low expression (blue line) of *IL22* based on Youden index. The probability of TRM in dependence of the months after biopsy is shown.

Supplementary Figure 3 Immunofluorescence staining of IL-22 and CD3 in the sigmoid colon biopsies of GvHD patients after allogeneic SCT. One of four representative biopsies derived from four independent patients is shown. The exemplified biopsy was taken on day 303 after SCT. The patient was not under steroids or Abx at the time of biopsy. IL-22 corresponds to AF488 (green) while CD3 corresponds to AF594 (red). Nucleus is counterstained with DAPI (blue signals). Image in the upper panel is taken at a 10× magnification. Lower panels represent magnified sections where white arrows represent CD3^-^IL-22^+^ cells and yellow arrows represent CD3^+^IL22^+^ cells. Scale bar, upper panel: 200 µm, lower panels: 50 µm.
