# Supplementary figures and images for: Low Intestinal IL22 Associates With Increased Transplant-Related Mortality After Allogeneic Stem Cell Transplantation
Source: Front Immunol. 2022 Apr 29;13:857400. doi: 10.3389/fimmu.2022.857400 (PMC9103485; doi:10.3389/fimmu.2022.857400)

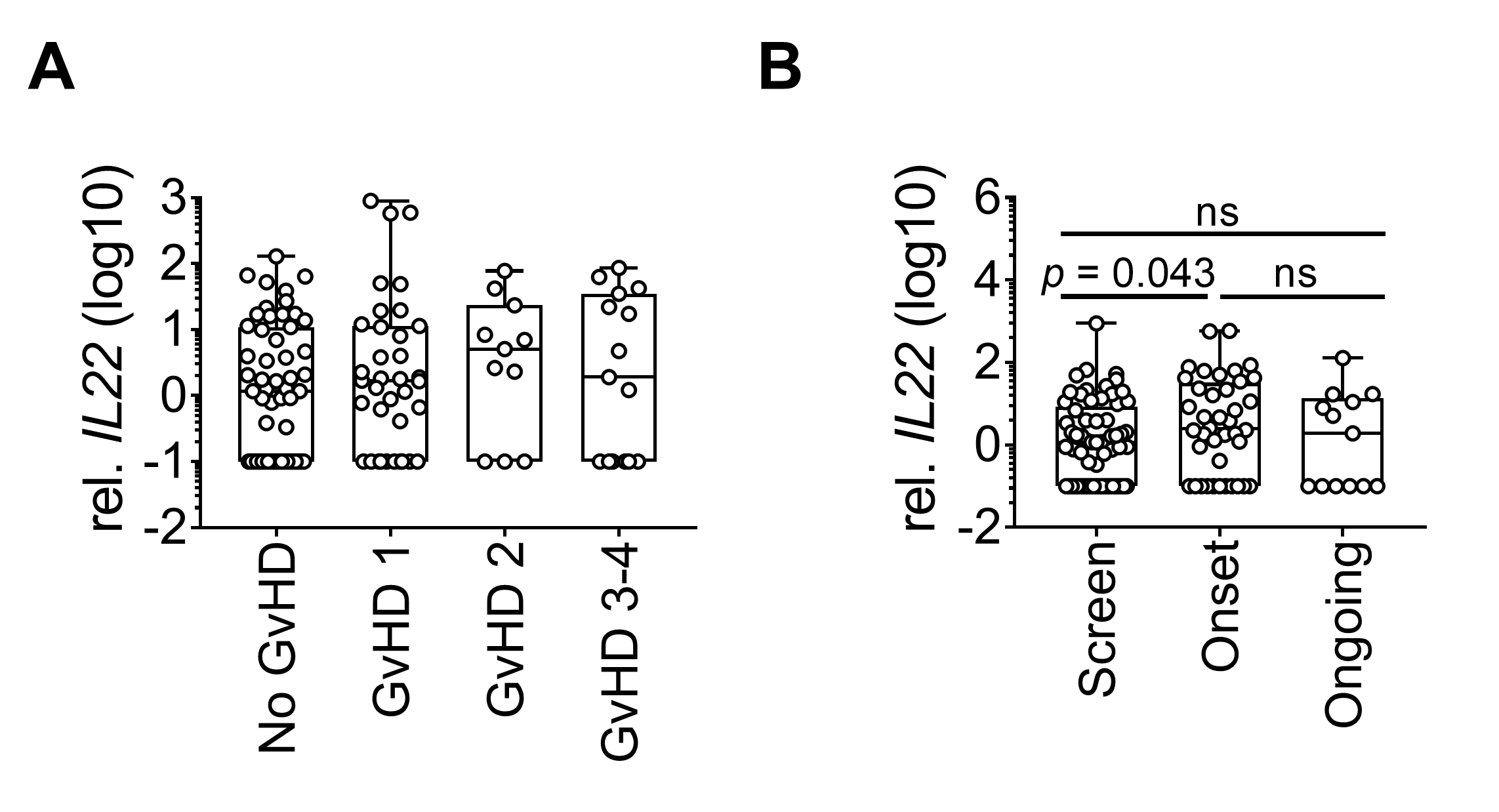

Supplement: Supplementary Figure 1 — IL22 expression in dependence of GvHD IL22 gene expression normalized to 18s rRNA was measured. (A) Patients were grouped according to histological GI-GvHD using Lerner`s grading system (no GvHD: n = 56, GvHD 1: n= 36, GvHD 2: n= 11, GvHD 3-4: n = 15), (B) Expression of IL22 in patients during clinical GvHD (screen: n = 65, onset: n = 40, ongoing: n = 13). Box plot diagrams depict median, upper, and lower quartiles and whiskers indicate minimal and maximal values. Negative values were set to 0.1. Statistical testing was performed by Mann-Whitney U test. ns, not significant. [file Image_1.tif]

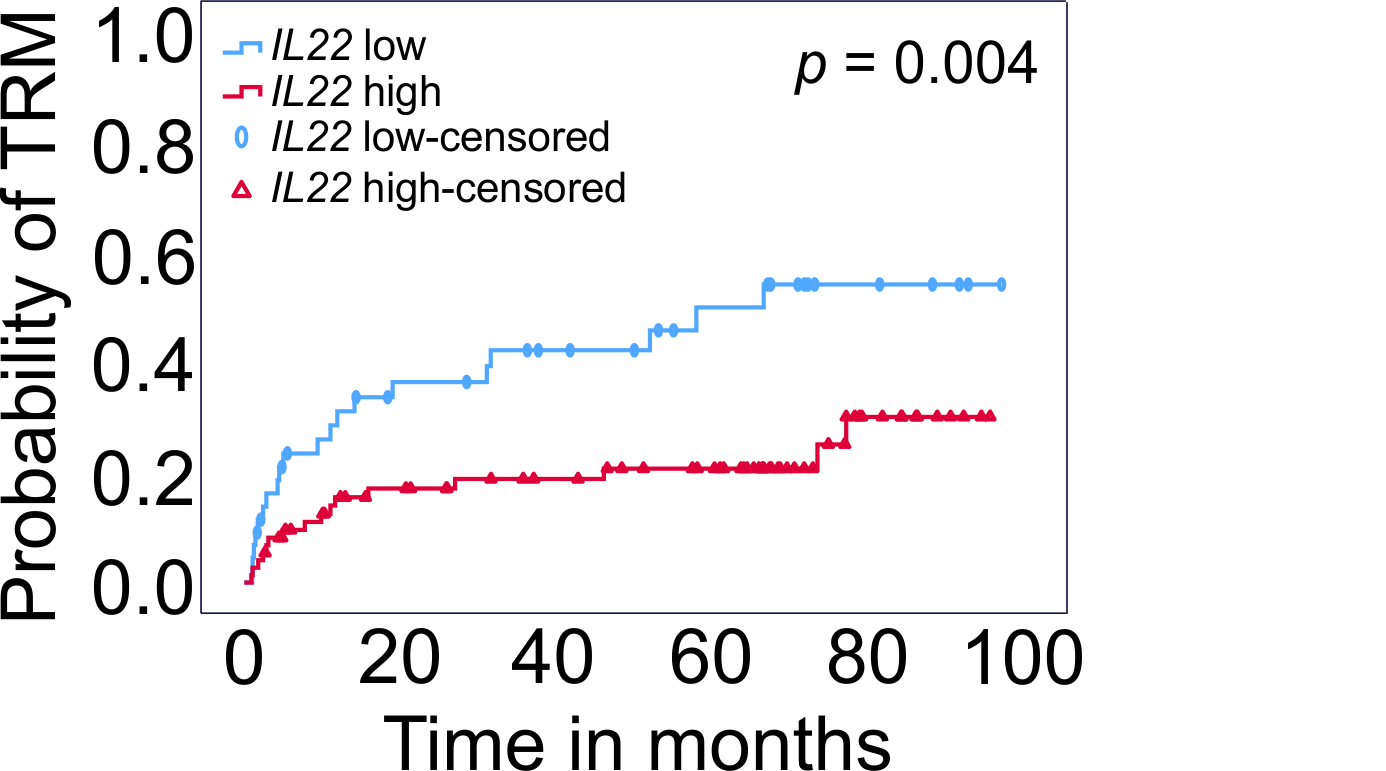

Supplement: Supplementary Figure 2 — Association of IL22 with probability of TRM. Patients were dichotomized in accordance to high (red line) and low expression (blue line) of IL22 based on Youden index. The probability of TRM in dependence of the months after biopsy is shown. [file Image_2.tif]

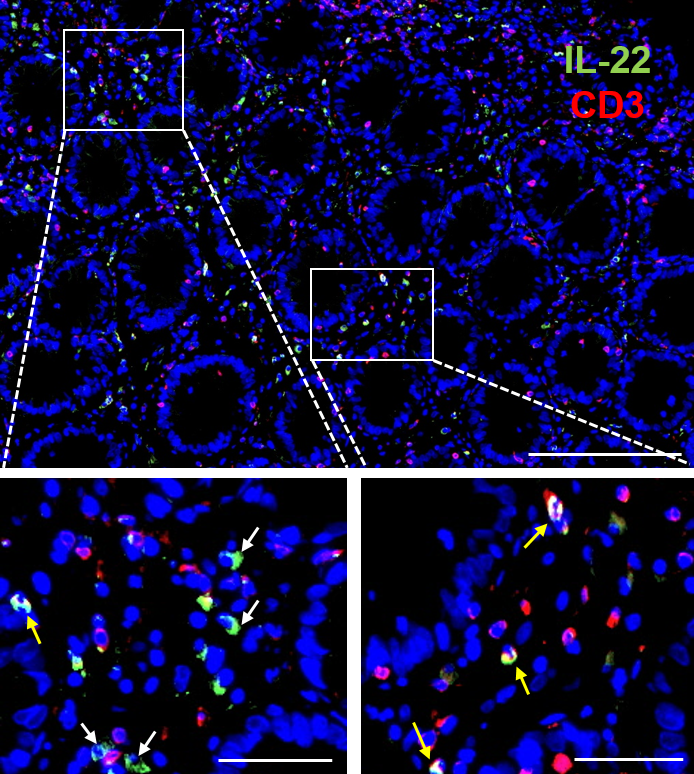

Supplement: Supplementary Figure 3 — Immunofluorescence staining of IL-22 and CD3 in the sigmoid colon biopsies of GvHD patients after allogeneic SCT. One of four representative biopsies derived from four independent patients is shown. The exemplified biopsy was taken on day 303 after SCT. The patient was not under steroids or Abx at the time of biopsy. IL-22 corresponds to AF488 (green) while CD3 corresponds to AF594 (red). Nucleus is counterstained with DAPI (blue signals). Image in the upper panel is taken at a 10× magnification. Lower panels represent magnified sections where white arrows represent CD3-IL-22+ cells and yellow arrows represent CD3+IL22+ cells. Scale bar, upper panel: 200 µm, lower panels: 50 µm. [file Image_3.tif]
